# Supplementary material for: Quantum efficiency of the B-center in hexagonal boron nitride
Source: Nanophotonics. 2024 Sep 27;14(11):1715–20. doi: 10.1515/nanoph-2024-0412 (PMC12133246; doi:10.1515/nanoph-2024-0412)
Supplement: Supplementary file 1 — Supplementary Material Details [file j_nanoph-2024-0412_suppl_001.docx]

**Supplemental information for “Quantum Efficiency Estimation of the B centres in hexagonal boron nitride”**

Karin Yamamura, Nathan Coste, Helen Zhi Jie Zeng, Milos Toth, Mehran Kianinia and Igor Aharonovich

*^1^ School of Mathematical and Physical Sciences, University of Technology Sydney, Ultimo, New South Wales 2007, Australia*

*^2^ ARC Centre of Excellence for Transformative Meta-Optical Systems, University of Technology Sydney, Ultimo, New South Wales 2007, Australia*

In this document, we provide supplementary information to “Quantum Efficiency Estimation of the B centres in hexagonal boron nitride”. It consists of two parts: (I) Optical measurement for confirmation of emitter wavelength, singleness and lifetime of B-centres (SI1-4), and (II) Estimation of the QE of B-centre in thick flake (SI5).

**I. Optical measurement of B-centres**

Figure SI1 shows the PL spectra of seven activated B-centres in hBN. The spectra were acquired under the 402 nm pulsed laser at 50 uW for 30 seconds. All emitters show the typical B-centre emission from ZPL at 436 nm. After confirming the emission wavelength, auto-correlation measurement was performed on the same emitters under a 402 nm pulsed laser at 50 uW with a repetition rate of 40 MHz. All emitters show the nature of singleness with g2(0) values ranging from 0.15 to 0.26 (shown in Figure SI2). Figure SI3 shows the confocal PL map of *the pre-characterized B-centres after transfer of the top hBN flake. The pre-characterised B-centres remain clearly visible since hBN is transparent and does not exhibit PL emission prior to electron beam irradiation. The measured emitters E1- E6 are shown in circles, E7 is located out of the PL map range.*

The lifetime measurements of these B-centres were measured before and after transferring the thick hBN on top of the hBN with B-centres (shown in Figure SI4). The average lifetime of B-centre in thin hBN flake ranges from 2.26 ns to 2.58 ns before transferring the top thick flake, and 1.73 ns to 2.26 ns after transfer.


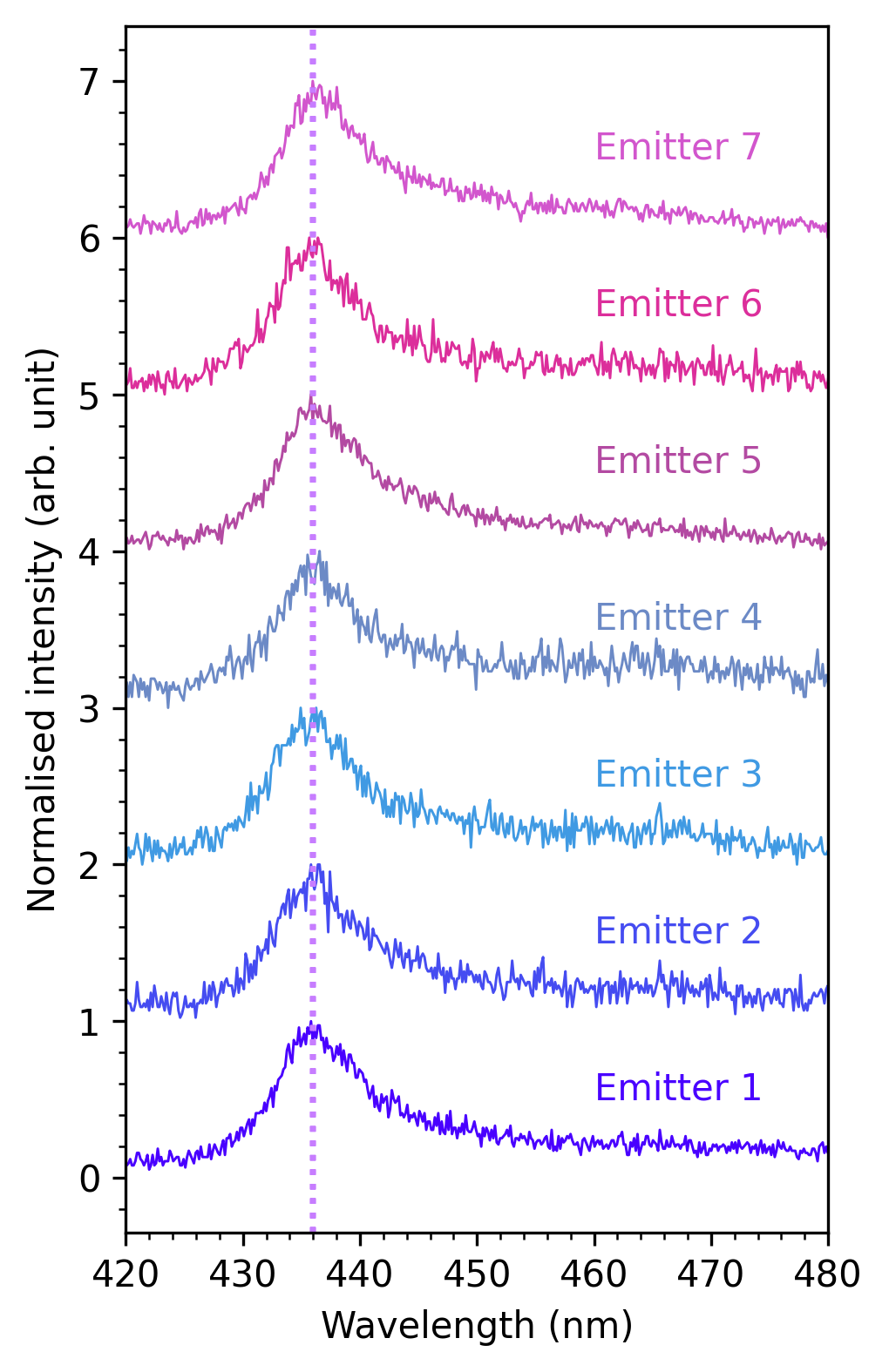


*Fig SI1. (a-g) PL spectra of seven investigated B centres in thin hBN.*


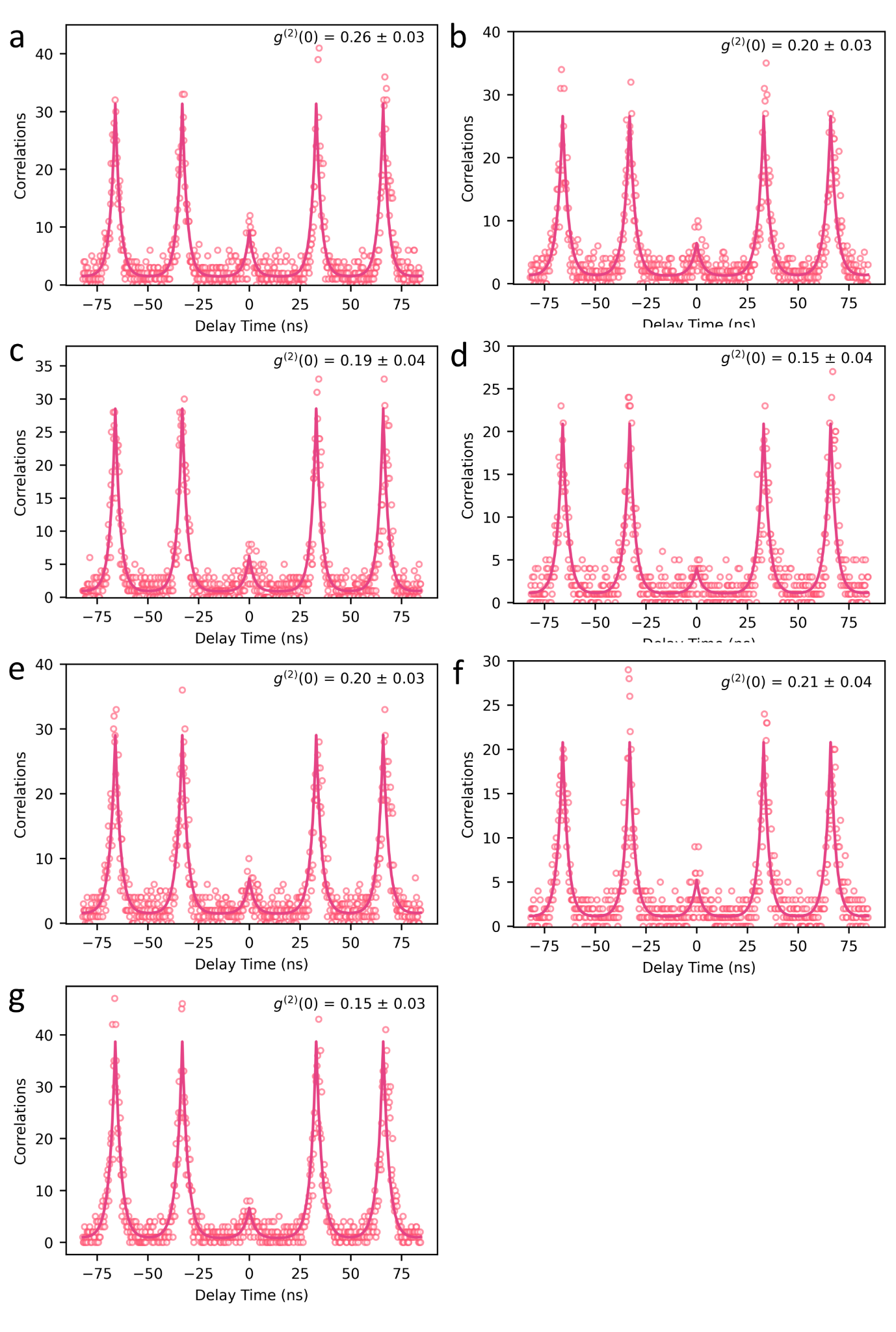


*Fig SI2. (a-g) Second-order correlation measurement on the same seven B-centres under pulsed laser excitation.*


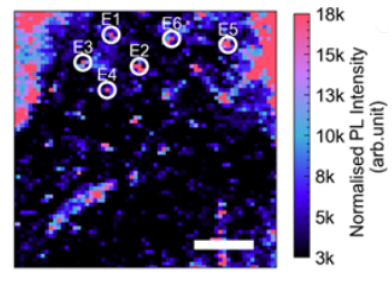


*Fig SI3. Confocal PL map showing an array of B-centres after transferring the top hBN. Emitter positions are labelled E1- E6. The scale bar corresponds to 5 μm.*


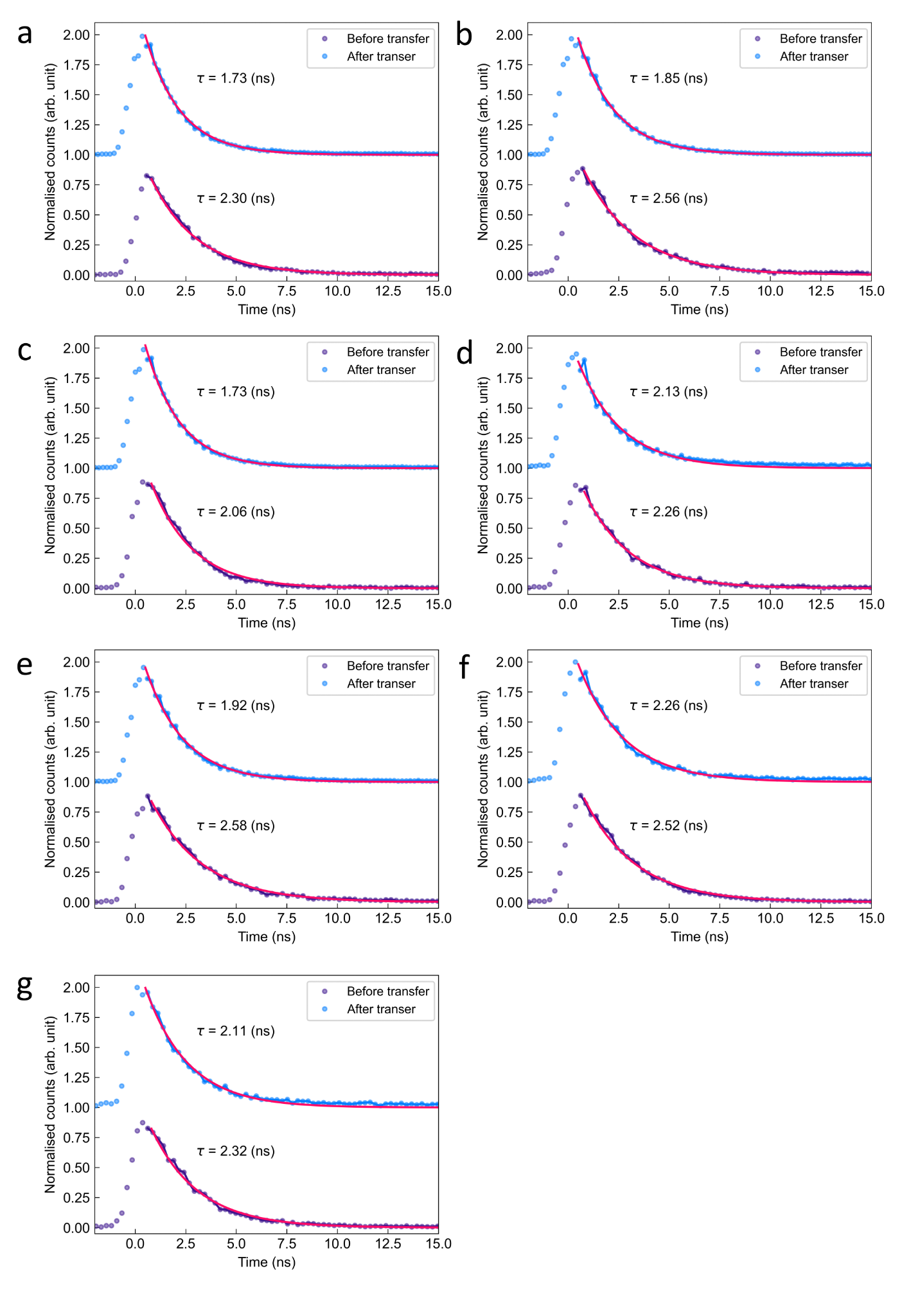


*Fig SI4. (a-g) Lifetime measurement of measured seven B centres in thin hBN before (light blue dots) and after (purple dots) the addition of the top hBN flake.*

**(II) Estimation of the QE of B-centre in thick flake**

Figure SI5 shows the emission saturation curve for an emitter in the same flake as previously studied and for an emitter in a thick (250 nm height) hBN flake for reference. The experimental data were fitted using the equation:

$I(P) = \frac{I_{sat}\cdot P}{P_{sat} + P}$ (SI E1)

We observe a large difference in count rate at saturation, with almost a factor 10 brighter emission for the thicker flake. Emitters in thin and thick flakes were bleached at excitation power of 850 μW and 3500 μW, respectively.

From these data, taken under the same experimental conditions, the internal QE of the thick flake could be deduced from the previously measured QE in the thin flake, by calculating the total system efficiency SE at saturation (including excitation, collection and detection efficiency):

$SE =\frac{1}{QE_{thin}}\cdot\tau_{thin}\cdot Counts (saturation)_{thin}$ (SI E2)

The QE in the reference thick flake can then be obtained by applying :

$QE_{thick} = Counts(saturation)_{thick}\cdot\tau_{thick}\cdot\frac{1}{SE}$ (SI E3)

With $\tau_{thin}$ and $\tau_{thick}$the measured lifetimes in the thin and reference thick flakes respectively. However, this method yields a QE value above unity. This is due to additional effects influencing the brightness, in particular the blinking of the emitter in the thin flake on a micro- to millisecond timescale[^1^](https://www.zotero.org/google-docs/?gfZDlJ), due to the proximity of emitters to the substrate[^2,3^](https://www.zotero.org/google-docs/?VbwpbZ). Characterising the blinking, especially at saturation power is challenging due to the bleaching of the emitter, however, performing the measurement after transferring the thin hBN flake on top of the hBN buffer, or on a suspended hBN membrane, could provide a solution to mitigate the effect of blinking and measure a more precise value of QE for emitters deep into the material.

*
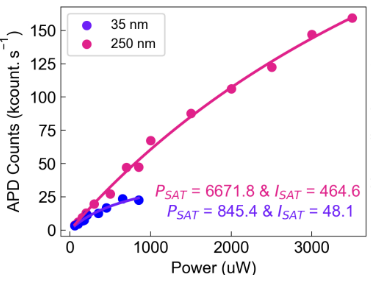
*

*Fig SI5.* *Emission saturation measurements of single B-centres in 35 nm and 250 nm thickness reference hBN.*

**References**

[1. Nedić, S., Yamamura, K., Gale, A., Aharonovich, I. & Toth, M. Electron Beam Restructuring of Quantum Emitters in Hexagonal Boron Nitride. *Adv. Opt. Mater.* **n/a**, 2400908.](https://www.zotero.org/google-docs/?lBeKSp)

[2. Li, X. *et al.* Nonmagnetic Quantum Emitters in Boron Nitride with Ultranarrow and Sideband-Free Emission Spectra. *ACS Nano* **11**, 6652–6660 (2017).](https://www.zotero.org/google-docs/?lBeKSp)

[3. Stern, H. L. *et al.* Spectrally Resolved Photodynamics of Individual Emitters in Large-Area Monolayers of Hexagonal Boron Nitride. *ACS Nano* **13**, 4538–4547 (2019).](https://www.zotero.org/google-docs/?lBeKSp)
